# The rising trend of papillary carcinoma in thyroidectomies: 14-years of experience in a referral center of Turkey

**DOI:** 10.1186/1477-7819-12-34

**Published:** 2014-02-10

**Authors:** Selim Yigit Yildiz, Huseyin Berkem, Bulent C Yuksel, Hakan Ozel, Murat Kendirci, Suleyman Hengirmen

**Affiliations:** 1 Department of Surgery, Kocaeli Derince Training and Research Hospital, 41100 Kocaeli, Turkey; 2 First Department of Surgery, Ankara Numune Training and Research Hospital, Talatpasa Bulvari, 06100 Ankara, Turkey; 3 Edirne State Hospital, Edirne, Turkey

**Keywords:** Thyroid papillary carcinoma, Chernobyl nuclear accident, Radiation, Goiter, Endemic

## Abstract

**Background:**

During the past 25 years, the incidence of thyroid papillary carcinoma (TPC), especially the micropapillary subtype, has been increasing in different countries worldwide. The rise in the rate of thyroid malignancies were also determined in Turkey in the last two decades. This fact was attributed to the Chernobyl accident because Turkey is one of the affected countries by the radioactive fallout. The aim of this study was to assess the changes in the parameters of the thyroid and put forth the reasons in a 14-year period.

**Methods:**

The patient records, demographic and malignancy characteristics, and operations of 1,585 patients who had a thyroidectomy from 1996 to 2009 were reviewed retrospectively. The study was divided in two equal time periods for comparison of data.

**Results:**

A total of 216 thyroid carcinomas (13.6%) were diagnosed in the study period. There was a significant increase in the frequency of papillary (*P* <0.023) and micropapillary (*P* <0.001) carcinomas when the two different time periods were compared. The rate of follicular, medullary and other types of malignancies did not change. In the second period (2003 to 2009) of analysis, the rate of micropapillary carcinoma (*P* = 0.001) and within male (*P* = 0.031) and female (*P* <0.001) genders, application of total thyroidectomy (*p* = 0.029), and multicentric disease (*P* = 0.015) increased significantly. A slight decrease in the mean age of the whole number of patients and patients with papillary and micropapillary carcinomas (*P* >0.05) was observed. The increased number of TPC >10 mm was insignificant. Geographic region and age specific malignancy increase was not determined.

**Conclusions:**

Micropapillary carcinoma has become a dominant type of thyroid malignancy in Turkey. The main reasons of this transition were mandatory iodization and much higher application of total thyroidectomy in surgery. Improvement in healthcare and diagnostic techniques are the complementary factors. Due to its lack of molecular and genetic basis from the perspective of thyroid cancer, the Chernobyl disaster has lost its importance in Turkey.

## Background

The incidence of thyroid malignancy has been increasing in Turkey for the past two decades [1,2]. Probable reasons which give rise to this situation in Turkey are environmental factors such as endemic iodine deficiency [3] and the Chernobyl disaster, and changes in the treatment and diagnosis options of thyroid diseases. It is well known that iodine deficiency is the most important factor for benign and malignant diseases of the thyroid [4]. Follicular and other undifferentiated tumors are more common in iodine-deficient areas [5,6].

Turkey was one of the affected regions by the Chernobyl radioactive fallout and in the last 15 years thyroid papillary carcinoma (TPC) has become more common in Turkey [7-9]. The major effect of the Chernobyl accident on human health demonstrated so far is the considerable rise in the incidence of TPC, particularly in children [10,11]. Owing to the fact that radioactive pollution has an increasing effect of papillary carcinoma and lack of genetic and molecular proof of the Chernobyl disaster has become a continuously speculated event.

The aim of this study was to assess the changes in parameters of thyroid malignancy, determining the reasons in a 14-year period. For this purpose the results of thyroidectomies which were applied to 1,585 patients were analyzed in our center.

## Methods

We retrospectively analyzed the data of 1,585 consecutive patients who had received surgical treatment for thyroid diseases at the 1st Department of Surgery, Ankara Numune Training and Research Hospital from 1996 to 2009. Patients come to our institution, which is one of the most well known referral centers in central Turkey. Our center is located in the middle of Anatolia that was one of the endemic iodine-deficient areas before mandatory iodization.

Specific data that were gathered included demographics, surgical procedure, histopathologic examinations, postoperative complications, and mortality. Each patient was evaluated with a physical examination, routine blood and hormonal assays, thyroid ultrasound scan (USG), and T^99^ scintigraphy. Serum thyroglobulin measurement was done in malignant diseases. Fine needle aspiration cytology (FNAC) was the final step in the evaluation for nodular cases. All the patients were examined with indirect laryngoscopy by the otolaryngologist preoperatively. Bilateral total thyroidectomy (BTT), bilateral subtotal thyroidectomy (BST), and lobectomy were the preferred surgical techniques in the management of thyroid diseases in this study period. All patients with preoperative diagnosis of malignancy underwent BTT with or without neck dissection depending on whether there was lymph node metastasis.

All the thyroid tumors were classified histologically and staged according to WHO and AJCC/UICC, 2002 TNM system [12,13].

In postoperative routine control for thyroid function, patients were given a physical examination at the third month initially and subsequently every 6 months for the next 2 years. During the follow-up period, patients with thyroid malignancy were evaluated for possible persistent or recurrent disease with physical examination, monitoring of thyroglobulin levels, I^131^ whole body scanning, and radiographic and functional nuclear imaging.

The time of study was divided in two equal periods for assessing and comparing the rate of malignancies, surgical preferences, and other data: 1996 to 2002 and 2003 to 2009.

### Statistical analysis

All data were collected and analyzed using SPSS 15.0 software (SPSS Inc., Chicago, IL, USA). Data were compared by paired *t* test within groups and differences between groups were analyzed using the Mann–Whitney *U* test and chi-square test where applicable.

## Results

**Figure 1:**
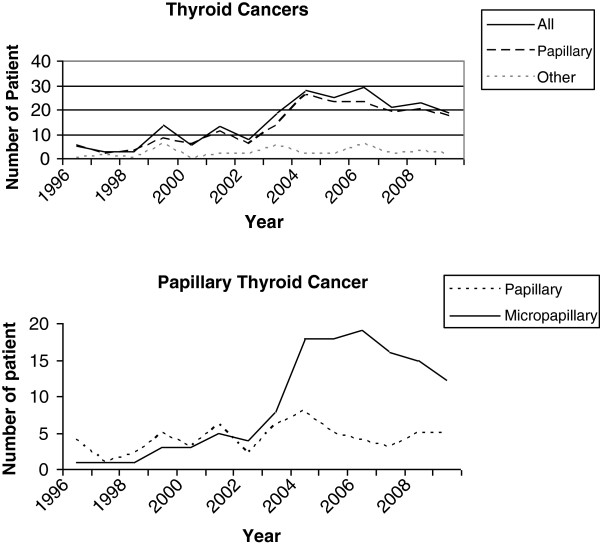
Changing trends of the thyroid cancers and thyroid papillary cancer between 1996 and 2009.

**Figure 2:**
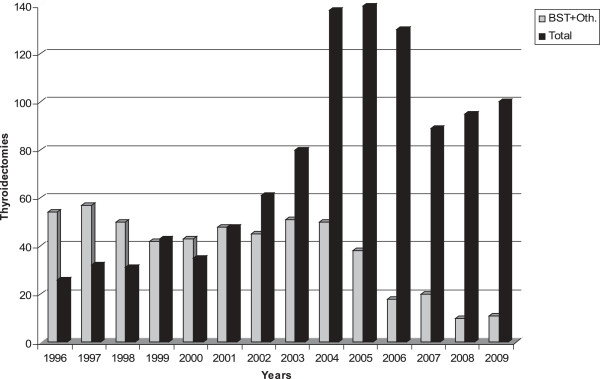
**Annual changes of thyroidectomy techniques.** Oth: Other.

A total of 1,585 patients with thyroid malignancy or different types of goiter underwent thyroidectomy. After the thyroidectomies it was found that 216 patients had thyroid carcinoma. The thyroid malignancy rate was 13.6% during the 14-year period. Demographic and other basic parameters of the study were shown in Table 1. Age and gender distribution of study periods were similar and no statistical difference was determined. Most of the thyroid malignancies were papillary carcinoma and these were found in 183 patients (84.7%). There was a significant increase in the frequency of total (52 *vs.* 164, *P* <0.021) and papillary carcinoma (41 *vs.* 142, *P* <0.023) between the time periods. The rate of follicular, medullary, and other (Hurtle cell, anaplastic, lymphoma, and metastatic) types of thyroid carcinomas had been reduced but this change not significant. How thyroid malignancies developed during the course of time was demonstrated in Figure 1. As presented annually in Figure 2, BTT has become a favorite surgical technique for several thyroid diseases and has taken the place of BST and lobectomy in the second period of study (273 *vs.* 776, *P* <0.029).

**Table 1:** Comparison the stages of study with different parameters

**Parameter**	**1996–2002**	**2003–2009**	***P *** **value**
N	618	967	
Age	42.7 ± 5.7	41.9 ± 3.8	
Gender (%)			
Male	128 (20)	205 (21)	
Female	490 (80)	762 (79)	
Carcinoma (%)	52 (8.4)	164 (16.9)	0.021
Papillary	41 (78.8)	142 (86.5)	0.023
Follicular	7 (13.4)	17 (10.3)	
Medullar	2 (3.9)	3 (2.5)	
Other	2 (3.9)	3 (2.5)	
Surgical operation (%)			
BTT	273 (44.1)	776 (80.2)	0.029
BST	302 (48.8)	141 (14.5)	0.032
Lobectomy	43 (7.1)	50 (5.3)	

Comparison of study periods (Table 2) for papillary carcinoma and micropapillary (pT <10 mm) subtype had been become the dominant form from 2003 to 2009 (Figure 2). There was a significant increase in the rate of micropapillary carcinoma (18 *vs.* 106, *P* <0.001) when TPC >10 mm frequency did not change. The mean age and diameter of primary tumor of the patients with papillary and micropapillary carcinoma decreased insignificantly. Under the age of 20 years, no malignancy rate increase was determined. Micropapillary carcinoma also increased in the male (*P* = 0.031) and female (*P* <0.001) genders after 2003. Multicentric disease increased (*P* = 0.001) in micropapillary subtype, when the ratio of lymph node metastasis did not change in both tumors. The number of other subtypes of papillary carcinoma did not change in 1996 to 2002 and 2003 to 2009. During the course of our study there was no significant increase in region-specific thyroiditis or thyroid cancer in patients from the Black Sea or other regions of Anatolia.

**Table 2:** Differences of thyroid papillary cancer parameters in time periods of study

	**Papillary**			**Micropapillary**		
**Parameter**	**1996–2002**	**2003–2009**	***P***	**1996–2002**	**2003–2009**	***P***
N	23	36	NS	18	106	0.001
Age	46.3 ± 3.2	45.2 ± 2.5	NS	45.7 ± 2.7	43.5 ± 3.9	NS
Gender						
Male	6	10	NS	6	20	0.031
Female	17	26	NS	12	86	0.001
Tumor size (cm)^a^						
T	2.5 ± 0.8	1.9 ± 0.6	NS	0.7 ± 0.2	0.6 ± 0.1	NS
Nodal metastasis						
N	8	11	NS	0	1	NS
Distant metastasis	0	0	NS	0	0	NS
Multicentric disease	5	8	NS	6	23	0.015
Subtype						
Classical	11	16	NS			
Follicular	10	17	NS			
Others	2	3	NS			

^a^Centimeter.

After the year 2003, more aggressive tumors were diagnosed. Three patients with follicular carcinoma were admitted with bone metastasis. During follow-up, five patients with malignancies died. Mortalities were caused by anaplastic (N = 3) and metastatic follicular carcinoma (N = 2).

## Discussion

The increasing trend in the incidence of thyroid carcinoma has been recognized for several decades among the neighbors of Turkey, in Europe, and distant countries [14-17]. There has been a marked increase in thyroid malignancies, especially papillary microcarcinoma (pT <10 mm), in Turkey during the last two decades [8,9].

In this present study undertaken in a 14-year period, the incidence of papillary and micropapillary carcinomas increased in patients with thyroidectomy. With respect to two equal time periods, thyroid micropapillary carcinoma had become dominant malignancy type in 2003 to 2009 (*P* <0.001). The frequency of follicular, medullary, and other type of thyroid malignancies had not changed.

The increase in the frequency of thyroid cancer could be attributable to several different reasons in the case of Turkey: the Chernobyl nuclear accident, mandatory iodization for endemic iodine deficiency, increased diagnostic scrutiny, strict pathologic investigation, and changing surgical techniques.

The importance of the Chernobyl accident is that it came to an end with life-threatening radioactive isotopes such as radioiodine fall-out. These isotopes (especially I-131) collected in the thyroid gland. After being exposed to radioactive isotopes, the risk of thyroid cancer occurrence is five to eight times higher in children aged under 5 years and young adults [18,19]. After the Chernobyl accident, increased prevalence of benign thyroid nodules and autoimmune thyroid diseases were reported [20]. In countries such as Ukraine and Belarus, where there had been severe pollution due to radiation, thyroid malignancies increased considerably, especially papillary carcinoma in children [21,22].

**Figure 3:**
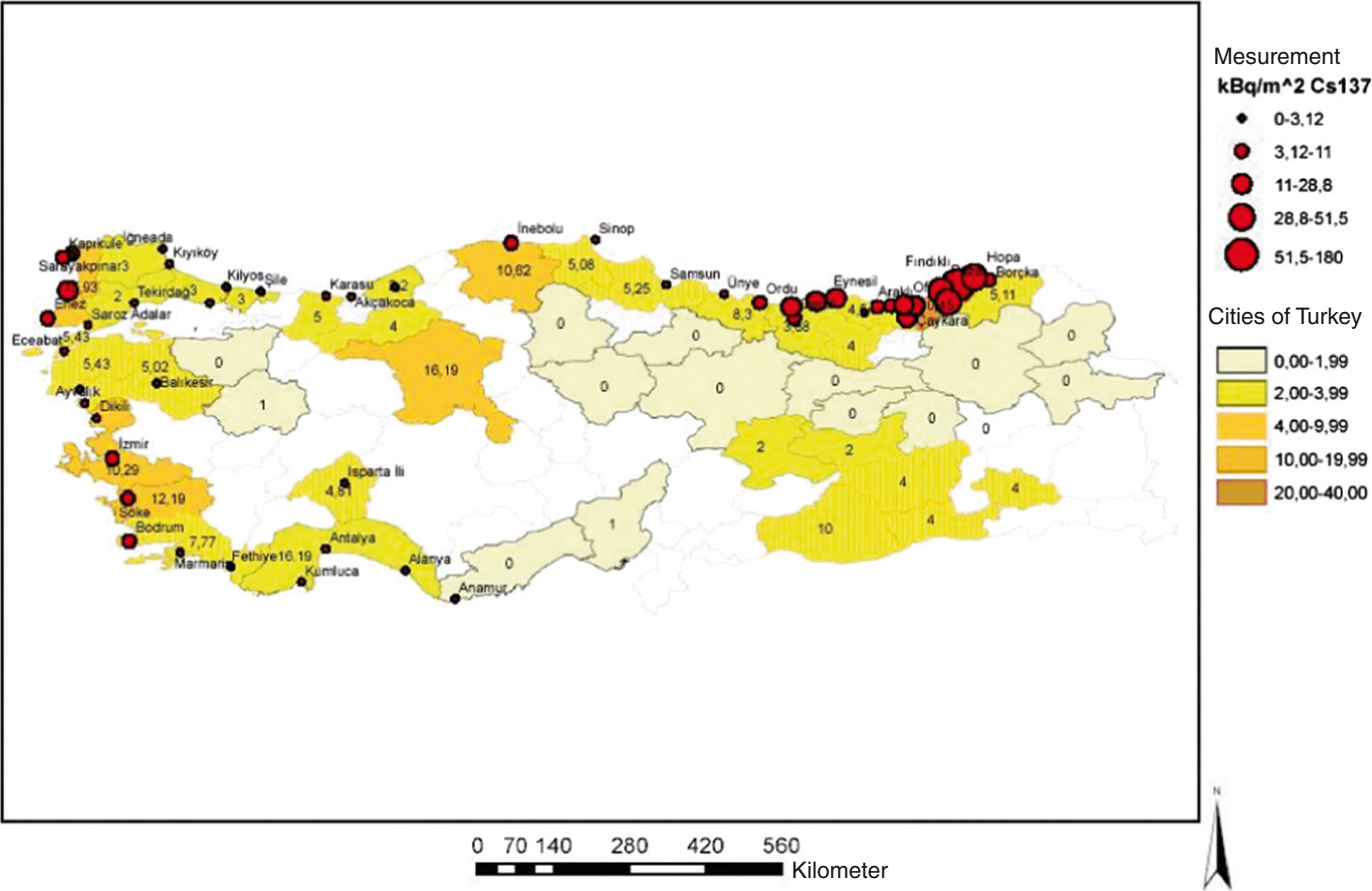
**Turkish Atomic Energy Authority’s **^**137**^**Cs surface soil measurement (as shown) in the map of Turkey in 1986.**

There has been much speculation as to how much radiation the Turkish population has been exposed to as a result of the Chernobyl nuclear power plant accident. It is a widely accepted fact that the north-eastern part of the Black Sea region of Turkey has been affected the most from the radioactive dispersion in 1986 [23]. Middle Anatolia was not seriously affected. After this nuclear accident, pollution with ionizing isotopes was shown with the measurement of the map of ^137^Cs in 1986 (Figure 3).

In 2003, Emral et al. [24] undertook a two-zone study in children who were aged under 5 years at the time of the Chernobyl accident and were living in the eastern Black Sea and inner Anatolia regions. This study pointed out that there was a slight increase in nodule prevalence and thyroid antibody-positive subjects in the Black Sea region. There was no malignant lesion determined in this region. During the course of our study there was no significant increase in region-specific thyroiditis or thyroid cancer in patients from the Black Sea or other regions of Anatolia. A slight decrease was established in the mean age of all cases and cases with papillary and micropapillary carcinomas in this present study. Under the age of 20 years, no malignancy rate increase was determined.

One other important factor is iodine deficiency which is endemic before 1999 in Turkey [3]. As of 1999, mandatory iodization of household salt has been put into effect in Turkey. Three years after this policy, iodine-deficient regions became iodine-sufficient areas [24]. It is known that both iodine deficiency and iodine excess have a triggering effect in thyroid malignancies, especially with regard to papillary microcarcinoma in the case of mandatory iodization [25,26]. This fact may have played a role in this study, because there was a marked increase in the rate of micropapillary tumors after 1999. In this study, reduction of the follicular and medullar carcinoma count was also determined but alteration of this rate was not significant.

One important point to take into consideration in this respect is that the socioeconomic status of the people and healthcare in Turkey which is more accessible due to the steadily increasing national income have significantly improved in the last 10 years. Changes in thyroid cancer incidence could also reflect increased use of diagnostic techniques such as USG and FNAC or changes in histological criteria [27,28]. At the end of the 1990s, WHO classification was applied more strictly in histopathologic investigations. In our study, increased frequency of micropapillary thyroid carcinoma also support this fact. Another important result of the study is in the case of histopathologic subtypes of papillary tumors. Most of the Chernobyl-induced papillary cancers were in solid-follicular subtype in mostly affected areas [29]. In this study micropapillary (*P* <0.001), classical, and follicular variants of papillary carcinoma were dominant.

Changes in the surgeons’ thyroidectomy technique was also established in this study. Surgeons prefer BTT after 2002 (*P* <0.029). The number of malignancies diagnosed, especially micropapillary carcinoma, has increased with much more excision of thyroid tissue by means of surgery. Multicentric disease was also higher (*P* = 0.001) due to total thyroidectomy. Increasing levels of thyroid malignancies, risks and difficulties of completion thyroidectomy, and problems of postoperative follow-up of thyroid cancers were the probable reasons of technique alteration. In surgical treatment of benign thyroid disesase today, BST is an acceptable technique internationally.

Molecular analyses are helpful for differentiating the radiation-induced thyroid carcinomas. Gene rearrangements over RET oncogene activation and mutations were common in post-Chernobyl papillary carcinomas in children [29,30]. In 2010, a study of Turkish Health Ministry was finalized [31]. This investigation was aimed at the effects of the Chernobyl accident on the 24th year on the health of the Turkish population. According to this study, the incidence of general cancer has increased in Turkey in the last 20 years but there has not been any region-specific advance. In the molecular analysis of thyroid cancers in the eastern Black Sea region, no radiation-induced DNA abnormality or mutations were determined.

## Conclusions

Thyroid malignancies, particularly papillary microcarcinoma, have been increasing in the last two decades in Turkey as in other regions of the world. Mandatory iodization and application of total thyroidectomy in surgery have become major reasons of thyroid cancer transition in Turkey in the last two decades. Improvements in healthcare and diagnostic techniques are the complementary factors. The dramatic effect of the Chernobyl accident on the increase of thyroid cancers is out of date. Molecular and genetic results do not support the detrimental effects of radiation.

## Abbreviations

AJCC/UICC: American Joint Cancer Committee/Union Internationale Contre le Cancer; BST: Bilateral subtotal thyroidectomy; BTT: Bilateral total thyroidectomy; FNAC: Fine needle aspiration cytology; THC: Thyroid papillary carcinoma; USG: thyroid ultrasound scan; WHO: World Health Organization.

## Competing interests

The authors declare that they have no competing interests.

## Authors’ contributions

SYY: Conception and design of study, interpretation of data. HB and BCY: Acquisition of data and design of study. HO and MK: Data collection and analysis, SH: Revision of the manuscript for important intellectual content. All authors read and approved the final manuscript.
